# Adolescents’ Social Network Site Use, Peer Appearance-Related Feedback, and Body Dissatisfaction: Testing a Mediation Model

**DOI:** 10.1007/s10964-015-0266-4

**Published:** 2015-03-19

**Authors:** Dian A. de Vries, Jochen Peter, Hanneke de Graaf, Peter Nikken

**Affiliations:** 1 Amsterdam School of Communication Research (ASCoR), University of Amsterdam, P.O. Box 15791, 1001 NG Amsterdam, The Netherlands; 2 Rutgers WPF, P.O. Box 9022, 3506 GA Utrecht, The Netherlands; 3 Netherlands Youth Institute, P.O. Box 19221, 3501 DE Utrecht, The Netherlands

**Keywords:** Social media, Body image, Gender, Peer influence, Appearance ideals, Adolescence

## Abstract

Previous correlational research indicates that adolescent girls who use social network sites more frequently are more dissatisfied with their bodies. However, we know little about the causal direction of this relationship, the mechanisms underlying this relationship, and whether this relationship also occurs among boys to the same extent. The present two-wave panel study (18 month time lag) among 604 Dutch adolescents (aged 11–18; 50.7 % female; 97.7 % native Dutch) aimed to fill these gaps in knowledge. Structural equation modeling showed that social network site use predicted increased body dissatisfaction and increased peer influence on body image in the form of receiving peer appearance-related feedback. Peer appearance-related feedback did not predict body dissatisfaction and thus did not mediate the effect of social network site use on body dissatisfaction. Gender did not moderate the findings. Hence, social network sites can play an adverse role in the body image of both adolescent boys and girls.

## Introduction

Body image plays an important role in adolescent development and wellbeing (Markey 2010). The body changes significantly during adolescence and adolescents need to cope with these changes (Markey 2010). Although many adolescents display some dissatisfaction with their bodies during this period, a high level of body dissatisfaction is a significant threat to adolescents’ wellbeing (Markey 2010). Adolescents who are more dissatisfied with their physical appearance are at an increased risk for suffering from, for example, depression, eating disorders, and low self-esteem (as reviewed by Markey 2010). As a result, the public and academics are keen to identify factors that contribute to body dissatisfaction (Markey 2010). The current study explores such a potential factor by investigating the effect of social network site use on body dissatisfaction.

The effect of social network sites on the lives and the development of adolescents is important to investigate given the role social network sites currently play in adolescents’ lives (O’Keeffe and Clarke-Pearson 2011). Social network sites are ubiquitous among adolescents: 70 % of European adolescents aged 14–17 use social network sites and 40 % of these users spend over 2 hours per day on these websites (Tsitsika et al. 2014). Social network sites consist of personal profiles of users (Pempek et al. 2009). Users present oneself to others on these profiles through text and pictures, they view and comment on the self-presentations of other users, and they read others’ comments on the own self-presentations (Espinoza and Juvonen 2011; Pempek et al. 2009). Personal photographs and physical appearance play an important role in these social network site activities (Ringrose 2011; Siibak 2009). Therefore, researchers have started to ask whether social network sites impact body image (e.g., Tiggemann and Miller 2010; Tiggemann and Slater 2013).

We currently know little about the effect of social network sites on body image. In two correlational studies, adolescent girls (aged 13–18 and 13–15) who used social network sites more frequently were more dissatisfied with their bodies (Tiggemann and Miller 2010; Tiggemann and Slater 2013). However, at least three important gaps remain in our knowledge about the relationship between the use of social network sites and adolescents’ body image. First, existing research on the effects of social network site use on body image is generally limited to cross-sectional data (Tiggemann and Miller 2010; Tiggemann and Slater 2013). These studies show that individuals who are more dissatisfied with their appearance *at the same time* use social network sites more frequently. However, we do not know whether social network site use is related to *changes* in adolescents’ body dissatisfaction over time (Tiggemann and Miller 2010; Tiggemann and Slater 2013). The first aim of the current study is therefore to test if frequency of social network site use predicts changes in body dissatisfaction among adolescents over time.

Second, we currently do not know which mechanisms explain the initial association found between social network site use and body image (Tiggemann and Miller 2010; Tiggemann and Slater 2013). In previous research, media use was indirectly associated with body dissatisfaction through peer influence (Clark and Tiggemann 2006). Girls aged nine to 12 who were exposed to appearance-focused TV and magazines more frequently at the same time reported having conversations about appearance with peers more frequently, which, in turn, was related to greater body dissatisfaction (Clark and Tiggemann 2006). Other studies have highlighted the impact that peers have on the body image of adolescents and adults of different ages (Eisenberg et al. 2003; Jones et al. 2004; Thompson et al. 1999a). We currently do not know if the use of social network sites also influences peers’ exchanges about physical appearance and if social network sites also impact adolescents’ body dissatisfaction indirectly through peer influence. The second aim of this study is therefore to test whether the use of social network sites affects body dissatisfaction indirectly through peer influence in the form of increased reception of peer appearance-related feedback.

Third, research on the effect of social network site use on body image has predominantly focused on girls (Tiggemann and Miller 2010; Tiggemann and Slater 2013). We understand this focus on girls because body image problems are more prevalent among adolescent girls than boys (O’Dea and Caputi 2001). However, adolescent boys also experience body dissatisfaction (McCabe and Ricciardelli 2001). Furthermore, some factors that affect girls’ body image also impact boys’ body image (as reviewed by Ricciardelli and McCabe 2004). The third aim of our study is therefore to investigate the relationships between social network site use, peer appearance-related feedback, and body dissatisfaction among both boys and girls and to establish if and how these factors and processes differ between boys and girls.

The tripartite influence model of body image, which is also referred to as the sociocultural model of body image, is a useful conceptual framework for investigating body image (Thompson et al. 1999b). This model describes that a variety of sociocultural channels, notably individuals’ parents, peers, and the media, convey beauty ideals to individuals (Thompson et al. 1999b). According to this model, individuals internalize these beauty ideals, and, to the extent that their own appearance does not match these ideals, become dissatisfied with their appearance (Thompson et al. 1999b). As most people do not think that they look like the ideal (Jacobi and Cash 1994), the vast majority of individuals will become more dissatisfied with their bodies when they evaluate the degree to which they match the appearance ideals (Thompson et al. 1999b). In this way, sociocultural channels contribute to body dissatisfaction.

Research among adolescents aged 10–15 has supported the notion that the media, parents, and peers influence adolescents’ body dissatisfaction (Keery et al. 2004; Shroff and Thompson 2006; Stanford and McCabe 2005) in line with predictions of the tripartite influence model (Thompson et al. 1999b). Studies that dealt with the effects of media, such as TV and magazines, have typically focused on the effects of exposure to highly idealized images of physically attractive people and appearance-focused content on body dissatisfaction [for meta-analyses see Bartlett et al. 2008 (males); Groesz et al. 2002 (females)]. However, few studies in the field of body image have paid attention to the changing media landscape and the rise of online media, such as social network sites (for notable exceptions see Meier and Gray 2013; Tiggemann and Miller 2010; Tiggemann and Slater 2013). The current study investigates if and how social network sites impact body image among adolescent boys and girls using the tripartite model of influence on body image as a framework.

## The Current Study

Initial research into adolescents’ activities and experiences on social network sites suggests that social network site use exerts a sociocultural influence on adolescents’ body image (Ringrose 2011; Siibak 2009). Physical appearance plays a central role in adolescents’ activities and experiences on different social network sites (Ringrose 2011; Siibak 2009). Social network site activities at least partly revolve around personal photographs (Espinoza and Juvonen 2011). When reporting on their most common social network site activities, 60 % of adolescents aged 12–13 named adding pictures to their profiles and 46 % named looking at other’s pictures (Espinoza and Juvonen 2011). Furthermore, adolescents choose pictures to upload to their social network site profile at least partly based on their physical appearance in the photograph (Siibak 2009). Among adolescents aged 11–18, 56 % of girls and 31 % of boys report good looks as the most relevant factor for choosing a picture to upload to their social network site profile (Siibak 2009). Furthermore, adolescent girls aged 14–16 report in interviews that they are very concerned with their physical appearance on social network sites (Ringrose 2011). These girls edited their photos to create a physically attractive representation of themselves on their profile and often received comments on their looks (Ringrose 2011).

According to the tripartite influence model (Thompson et al. 1999b), sociocultural influences to look attractive contribute to body dissatisfaction, as individuals generally do not meet the sociocultural beauty ideals (Jacobi and Cash 1994). If, as previous research suggests, social network site use exerts a sociocultural influence on body image (Ringrose 2011; Siibak 2009), the tripartite model would thus predict that social network site use will lead to body dissatisfaction. In line with the notion that social network site use exerts a negative influence on body image, some studies have initially shown that social network site use is negatively related to body image among adolescent girls aged 13–18 (Tiggemann and Miller 2010; Tiggemann and Slater 2013). Based on the tripartite influence model (Thompson et al. 1999b) and the evidence that social network sites exert a sociocultural influence on body image (Ringrose 2011; Siibak 2009), the most likely explanation of this correlation is that social network site use leads to increased body dissatisfaction. We, therefore, hypothesized that more frequent social network site use predicts increased body dissatisfaction among adolescents (H1).

The use of appearance-focused media can also impact the degree of appearance-focus in adolescents’ peer-exchanges. The use of appearance-focused media may contribute to an “appearance culture” among peers (Jones et al. 2004), which includes talking about each other’s physical appearance and how to improve it. In line with this notion, research among girls aged nine to 12 shows that girls who more frequently use appearance-focused media (television and magazines) also have conversations about their appearance with their peers more often (Clark and Tiggemann 2006). Scholars have proposed that the appearance ideals that adolescents see in the media become personalized when they talk with peers (Clark and Tiggemann 2006; Jones et al. 2004). The use of appearance-focused media may, therefore, lead adolescents to more frequently receive appearance-related feedback from peers.

Because social network sites are also focused on appearance to a great extent (Ringrose 2011; Siibak 2009; Wang et al. 2010), the use of social network sites may also lead adolescents to more often exchange feedback on each other’s physical appearance. For example, adolescents may talk about the pictures they and others have posted on a social network site in later conversations with their friends. Peers may evaluate the physical appearance of themselves and others and exchange tips to improve their looks. Adolescents can exchange this peer appearance-related feedback both within and outside of the social network site platform. In this way, adolescents’ use of social network sites may lead them to receive peer appearance-related feedback more frequently. To our knowledge, previous research has not investigated whether social network site use indeed impacts appearance-related exchanges among peers in such a way. However, based on research on other appearance-related media (Clark and Tiggemann 2006), we hypothesized that more frequent social network site use predicts increased peer appearance-related feedback among adolescents (H2a).

According to the tripartite model, the reception of peer appearance-related feedback will negatively impact adolescents’ body image (Thompson et al. 1999b). Peer appearance-related feedback pressures adolescents to conform to an appearance ideal that they do not meet (Thompson et al. 1999b). Adolescents become aware of discrepancies between their own bodies and the appearance ideal and, as a result, become dissatisfied with their appearance (Thompson et al. 1999b). In line with this prediction, adolescents (aged 12–17) who received peer appearance-related feedback more frequently were less satisfied with their bodies (Eisenberg et al. 2003; Jones et al. 2004). Therefore, we hypothesized that more frequent reception of peer appearance-related feedback predicts greater body dissatisfaction among adolescents (H2b).

Media may impact adolescents’ body image indirectly through peer influence (Clark and Tiggemann 2006; Jones et al. 2004). Among girls aged nine to 12, exposure to appearance-focused magazines and TV was not related to body dissatisfaction directly, but only indirectly through appearance conversations with peers (Clark and Tiggemann 2006). The use of appearance-focused media (television and magazines) was related to more frequent conversations about appearance among peers, which was in turn related to body dissatisfaction (Clark and Tiggemann 2006). Social network sites may also impact body dissatisfaction indirectly through peer influence. If the use of social network sites increases peer appearance-related feedback (H2a), and peer appearance-related feedback increases body dissatisfaction (H2b), then peer appearance-related feedback will mediate the effect of social network site use on body dissatisfaction among adolescents. We, therefore, hypothesized an indirect effect of social network site use on body dissatisfaction through peer appearance-related feedback (H2c).

The hypothesized relationships between social network site use, peer appearance-related feedback, and body dissatisfaction may differ in strength depending on adolescents’ gender. The tripartite influence model emphasizes that adolescents’ body dissatisfaction results from sociocultural channels exerting pressures to conform to unrealistic beauty ideals (Thompson et al. 1999b). Although sociocultural pressures to conform to appearance ideals influence both boys and girls (aged 12–16), research suggests that these pressures impact girls to a greater extent than boys (Jones et al. 2004). One experiment among adolescents aged 13–18 found that exposure to idealized appearance in mass media contributed to body dissatisfaction among girls but not among boys (Hargreaves and Tiggemann 2004). Furthermore, there is evidence that girls are generally subjected to such pressures to a greater extent than boys, at least among adolescents aged 12 to 16 (McCabe and Ricciardelli 2001). Research thus indicates that sociocultural influences on body image disproportionally affect girls.

Males and females may also differ in the degree of pressure to conform to appearance ideals that they experience on social network sites and the resulting impact of these pressures on body image. Young adults pay more attention to females’ than males’ physical appearance on social network sites (Seidman and Miller 2013). Furthermore, adult users evaluate females more strongly based on their physical appearance than males on social network sites (Manago et al. 2008). These gender differences may also occur among adolescents. According to the tripartite model (Thompson et al. 1999b), if social network sites exert greater pressures to look attractive on girls than on boys, the use of these social network sites will influence the body image of girls to a greater extent than the body image of boys. We, thus, hypothesized that social network site use will lead to body dissatisfaction more strongly among adolescent girls than boys (H3a).

One way in which social network sites may exert greater influence on girls’ than boys’ body image is that social network site use may increase the reception of appearance-related feedback to a greater extent for girls than for boys. In general, adolescent girls receive comments about their physical appearance more often than boys do, at least at ages 12–15 (McCabe et al. 2006). Furthermore, if people pay more attention to females’ than males’ physical appearance on social network sites (Seidman and Miller 2013) and girls are evaluated more strongly based on their appearance on these websites (Manago et al. 2008), the use of social network sites likely instigates more peer appearance-related feedback targeted at girls than peer appearance-related feedback targeted at boys. We, therefore, hypothesized that the effect of social network site use on peer appearance-related feedback would be stronger among adolescent girls than boys (H3b).

The effect of appearance-related feedback from peers on body image may also depend on the gender of the receiver. For example, in a study among adolescents aged 11–18, girls were more bothered by appearance-related feedback than boys, at least when it concerned weight-related teasing (Neumark-Sztainer et al. 2002). As a result, appearance-related feedback may be more detrimental to girls’ than to boys’ body image. In line with this notion, a meta-analysis of research among children, adolescents, and adults has shown that appearance-related feedback affects females’ body image more negatively than males’ body image (Menzel et al. 2010). We, therefore, hypothesized that the effect of peer appearance-related feedback on body dissatisfaction would be stronger among adolescent girls than boys (H3c).

## Methods

### Sample and Procedure

A two-wave panel survey was conducted by the Netherlands Youth Institute (Nederlands Jeugdinstituut) and Rutgers WPF (Dutch Expert Centre on Sexuality). In addition to the measures described below, the survey also included questions related to (sexual) media use, sexual attitudes and behaviors, and body image (as published in Nikken and de Graaf 2013; de Vries et al. 2014). The first wave was conducted in July through September 2008 and the second wave in December 2009. The survey was conducted among children of members of Intomart GfK, an online access panel that consists of 25,000 members across the Netherlands. Recruitment across the Netherlands improves generalizability in comparison with convenience samples. Coverage bias due to the use of an online panel was unlikely because Internet access was 98 % among people under 25 in the Netherlands (Centraal Bureau voor de Statistiek 2012).

In all, 3,160 Intomart GfK members who were parents of at least one child aged between 11 and 18 were contacted with a screening questionnaire and asked for permission to contact their children. Of these members 50.6 % responded, gave permission, and filled out the screening questionnaire completely. As a result, 1,600 adolescents were invited to participate in the first wave, of whom 1,294 (80.9 %) completed the questionnaire. For the second wave, adolescents who had completed the first questionnaire were asked, again via their parents, to complete a questionnaire similar to the first. In total, 604 adolescents (50.7 % female) completed all measures that were of interest for the current study in both waves. Retention rate was thus 54.2 %.

The age of participants in the final sample ranged between 11 and 18 (*M* = 14.7, *SD* = 1.7 at time 1; 50.7 % female). In this sample 20.1 % of the boys and 18.0 % of girls were aged 11–12, 33.9 % of boys and 41.2 % of girls were aged 13–14, 33.9 % of boys and 29.4 % of girls were aged 15–16, and 12.1 % of boys and 11.4 % of girls were aged 17–18. With regards to ethnicity, almost all parents (97.7 %) of the included adolescents reported having been born in the Netherlands. With respect to BMI, of the participants who reported their weight and height (13.1 % did not), 98.9 % reported a BMI under 30, 91.4 % of participants reported a BMI under 25, 52.3 % reported a BMI under 20, and 30.0 % a BMI under 18. Regarding parental monitoring of online behavior, only 2.5 % of parents reported knowing nothing about what their child does on the Internet, whereas 48.8 % reported knowing a lot about this and the remaining 48.7 % reported knowing a little about this. With respect to pubertal status, 73.2 % of the boys had experienced that their voice changed. Of the girls 81.0 % reported that they had experienced menarche. With respect to socioeconomic status, the highest category (A) was assigned to 11.9 % of participants. The second highest category (B1) was represented the most frequently with 41.2 % of participants assigned to this category. A further 21.9 % of participants were in category B2 and 23.3 % of participants were in category C. Only 1.7 % of participants were in the lowest socio-economic level (D).

There were no differences in terms of gender, *t*(1292) = .082, *p* > .05, or level of education, *t*(1292) = −1.09, *p* > .05 between adolescents who completed both waves and those who dropped out after the first wave. However, respondents who only completed one wave were 4 months older, on average, than respondents who completed both waves, *t*(1292) = −3.32, *p* = .001. The sample did not deviate from official Dutch population statistics in terms of gender, but participants were more likely to receive higher levels of education and to have parents born in the Netherlands than average for the Dutch population (Centraal Bureau voor de Statistiek 2012).

### Measures

#### Social Network Site Use

Frequency of social network site use was assessed with the question: “How often did you visit Hyves.nl in the past 6 months?” The response options were 0 (*never*), 1 (*sometimes*), 2 (*regularly*), 3 (*often*), and 4 (*always*) (*M* = 2.4, *SD* = 1.5 at time 1; *M* = 2.6, *SD* = 1.4 at time 2). A single item was used as recommended for constructs that are concrete and singular (Bergkvist and Rossiter 2007). Although there were other social network sites on which some adolescents had a profile (e.g., MySpace), Hyves.nl was the dominant and most popular social network site at the time of the study (like Facebook is currently). Of adolescents aged 12–17, 75 % had a profile on the website (Mijn Kind Online 2009). The current survey also assessed the use of other social network sites that were around at the time of the survey. However, the use of these websites in this sample was negligible and, therefore, we only focused on popular social network site Hyves. Hyves.nl was comparable to Facebook in terms of its goal, set-up, and technological possibilities. Important for the current study is that on Hyves (like on Facebook, Instagram, Rate, and Bebo), posting personal photographs and comments on friends’ profiles played a central role (Mijn Kind Online 2009).

#### Peer Appearance-Related Feedback

Peer appearance-related feedback was measured using a scale constructed specifically for the survey. This scale consisted of four items asking participants how often their friends (1) give them tips to get a more beautiful body, (2) give them criticism about their appearance or clothes, (3) give them tips to look sexy, and (4) tell them it is important to look good. The response options ranged from 0 (*never*) to 4 (*very often*). Factor analysis showed that the four items could be combined into a single factor that explained 62.6 % of the variance. The items were averaged to create a composite score. Cronbach’s alpha was .79 at time 1 and .82 at time 2 (*M* = 0.53, *SD* = 0.57 at time 1; *M* = 0.59, *SD* = 0.60 at time 2).

#### Body Dissatisfaction

Body dissatisfaction was assessed using a version of the Dutch translation of the Body Areas Satisfaction Scale, a subscale of the Multidimensional Body-Self Relations Questionnaire (Cash 1994). This scale has been translated and successfully used among Dutch adult males and females (Woertman and van den Brink 2008). We used the formulations of the translation by Woertman and van den Brink (2008) and adapted it so that the specific body parts would be clearer for adolescents and could be used among both boys and girls. The scale consists of items that ask respondents how satisfied they are with eight different appearance attributes (face, hair, buttocks, stomach, breasts or chest, genitals, muscularity, and body weight). The response options ranged from 0 (*very satisfied*) to 4 (*very dissatisfied*). Scores were averaged to create a composite score. Factor analysis revealed that all eight items loaded on a single factor that explained 49.1 % of the variance. Cronbach’s alpha was .85 at time 1 and .84 at time 2 (*M* = 1.46, *SD* = 0.65 at time 1; *M* = 1.45, *SD* = 0.65 at time 2).

#### Additional Variables

In addition to the key variables, we also measured a number of additional variables at time 1 to provide more information on the sample. *BMI* was calculated as self-reported weight in kilograms divided by self-reported height in meters squared. BMI ranged from 13.59 to 36.73 (*M* = 20.04*, SD* = 3.54) in the current sample. *Parental monitoring of the adolescents’ online behavior* was reported by one of the parents. This parent answered the question “How much do you know about what your child does on the Internet.” This question could be answered with 0 (*I know nothing about this*), 1 (*I know a little about this*), or 2 (*I know a lot about this*) (*M* = 1.46*, SD* = 0.55). *Age* at time 1 was calculated from the self-reported birthdate and the date the survey was completed at time 1, and expressed in years (*M* = 14.75*, SD* = 1.67). *Pubertal status* was measured by asking the boys whether their voice had gotten lower and the girls whether they had experienced their first menstruation already. *Ethnicity* was assessed through parent-reported country of birth of this parent. *Socioeconomic status* of the family was measured using the Dutch gold standard used by Statistics Netherlands (Centraal Bureau voor de Statistiek 2012). This measure is based on the family breadwinner’s level of education and current occupation. Based on the parental responses, participants were categorized into five categories ranging from low (D) to high (A).

### Data Analysis

**Fig. 1:**
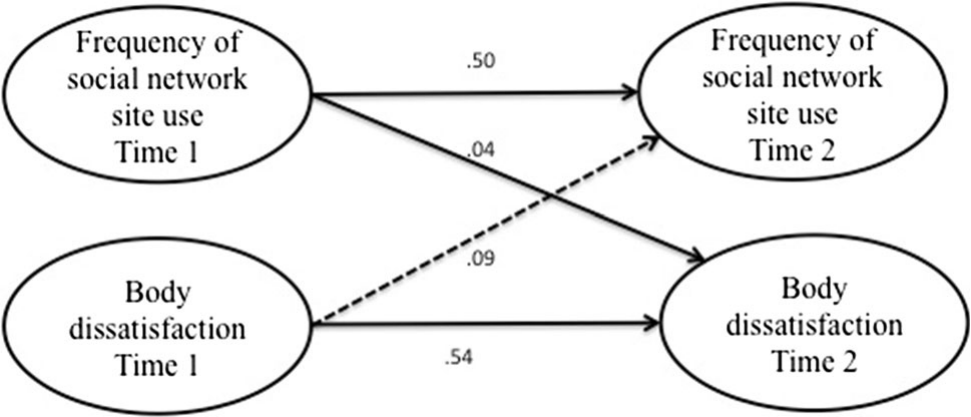
Simplified illustration of AMOS model of relationships between social network site use and body dissatisfaction. *Numbers* indicate unstandardized regression coefficients for paths. *Dashed* *lines* indicate that the represented path was not statistically significant

**Fig. 2:**
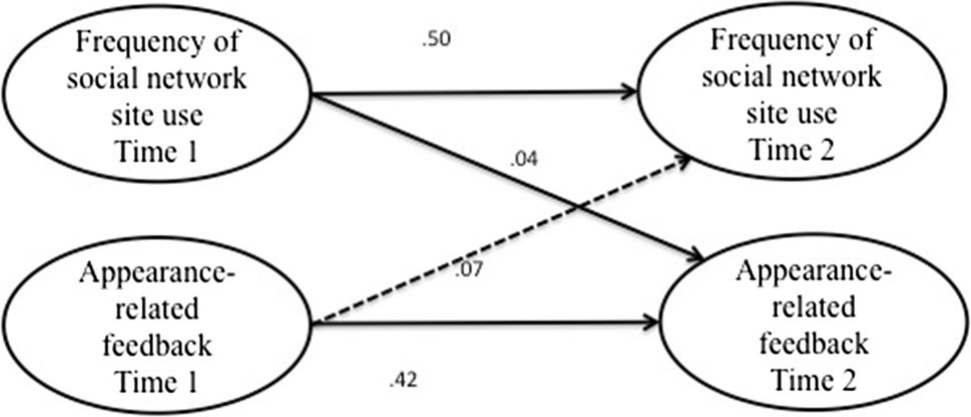
Simplified illustration of AMOS model of relationships between social network site use and peer appearance-related feedback. *Numbers* indicate unstandardized regression coefficients for paths. *Dashed* *lines* indicate that the represented path was not statistically significant

**Fig. 3:**
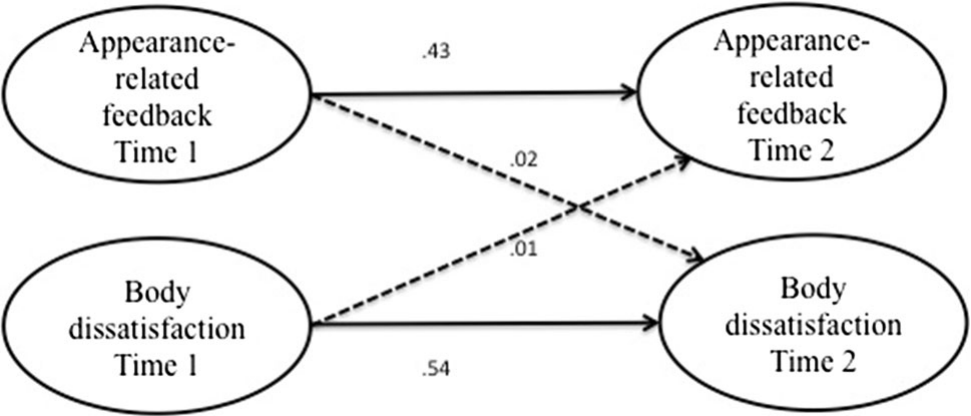
Simplified illustration of AMOS model of relationships between peer appearance-related feedback and body dissatisfaction. *Numbers* indicate unstandardized regression coefficients for paths. *Dashed* *lines* indicate that the represented path was not statistically significant

**Fig. 4:**
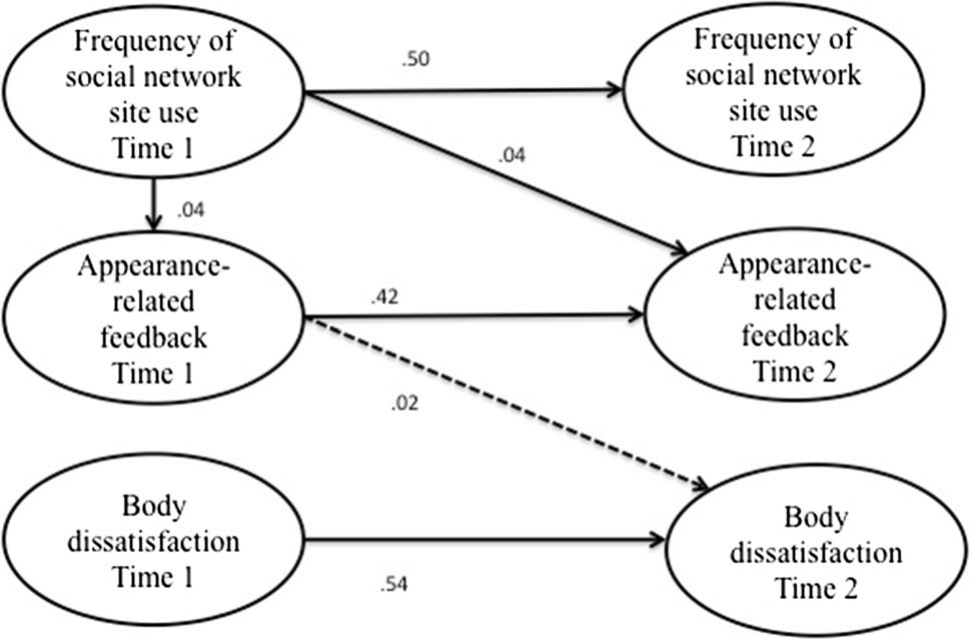
Simplified illustration of AMOS model of the indirect effect between social network site use, peer appearance-related feedback and body dissatisfaction. *Numbers* indicate unstandardized regression coefficients for paths. *Dashed lines* indicate that the represented path was not statistically significant

First, correlations were calculated between all measures at both time points for the sample as a whole and for boys and girls separately. Because the distributions of the scores on our key measures were skewed we calculated non-parametric correlations in Stata 12, namely Kendall’s tau-a, and converted this value to an approximation of Pearson’s *r* using Greiner’s relation to aid interpretation, as recommended by Newson (2002). Subsequently, we tested the first hypothesis and the second set of hypotheses in four separate models (see Figs. 1, 2, 3 and 4) using structural equation modeling in SPSS, AMOS version 19. To test the direction of effects hypothesized in H1, H2a, and H2b, we used three cross-lagged models (Figs. 1, 2 and 3), testing both the hypothesized effect and the effect in the opposing direction. Then we modeled the hypothesized mediation effect postulated in H2c using the mediation model displayed in Fig. 4. In order to test the hypothesized moderation of gender specified in the third set of hypotheses, the models were subjected to multiple group analysis.

In the set-up of the models, we followed recommendations by Cole and Maxwell (2003). All analyses therefore included previous levels of the variables of interest. In this way, we controlled for past behavior, which increases the validity of the influence of the predictor variables at time 1 on the outcome variables at time 2 (Cudeck 1991; Gollob and Reichardt 1991). In addition, all analyses controlled for gender, given the expected gender differences on the key variables. In the structural equation models, the eight items of the body dissatisfaction scale were combined into three parcels using the item-to-construct balanced procedure suggested by Little et al. (2002). Item parceling results in more parsimonious models and reduces the chance of double loadings and the influence of sampling error (Little et al. 2002). We included the single-item measure social network site use into the model as a manifest variable. The four items measuring peer appearance-related feedback were not subjected to item parceling, as there were not enough items to create three parcels, the recommended number in the item-to-construct balanced procedure (Little et al. 2002). Therefore, we included the four items as observed variables and loaded each item separately on the latent construct of peer appearance-related feedback.

The assumption of multivariate normality required for the traditional parametric tests was not met according to results of Shapiro–Wilk tests. In addition, Breusch-Pagan/Cook-Weisberg tests showed evidence of heteroscedasticity. To alleviate statistical problems due to violation of the assumption of normality we applied the bootstrap method to all models (1,000 bootstrap samples, *N* = 604 each) (Efron and Tibshirani 1993), and based our conclusions both on the bootstrap bias–corrected and accelerated 95 % confidence intervals as well as on the results of the parametric tests for the estimates. We only considered a hypothesis supported if the results of both tests were consistent. In addition, because parametric regression-based tests with heteroscedastic data can yield biased and inconsistent standard errors, resulting in Type 1 errors, we also conducted regression analyses with heteroscedasticity-consistent standard errors (hc3) in Stata 12, as recommended by Hayes and Cai (2007).

A number of variables may impact social network site use, peer appearance-related feedback, and/or body dissatisfaction: BMI, parental monitoring of the adolescent’s online behavior, age, pubertal status, and socioeconomic status. These variables may thus act as third variables in the hypothesized models. As our models control for past behavior of each outcome, the models are expected to be fairly robust against third variable effects. However, as confounding effects may still occur, we also ran the models (Figs. 1, 2, 3 and 4) controlling for these factors. The control factors were entered in the model as manifest variables, covaried with the predictor variables, and paths representing the influence of the control factors on the outcome variables were drawn. The results are not included in the body of text of the results section, as we had not formulated specific hypotheses about the impact of these variables. Moreover, AMOS does not allow for analyses with missing cases and including the covariate BMI would result in a smaller and biased sample (525/604) because some adolescents did not report their height or weight. We provide information regarding these additional analyses in footnotes 1–4.

## Results

### Descriptive Statistics and Correlations

As shown in Table 1, girls visited social network sites more frequently, experienced peer appearance-related feedback more often, and were more dissatisfied with their bodies than boys. In addition to what is displayed in Table 1, it is interesting to note that, at time 1, 58.1 % of the boys and 79.1 % of the girls visited the social network site “regularly” to “always.” At time 2, this was respectively 66.5 % and 87.3 %. In contrast, at time 1, 25.5 % (time 2: 19.8 %) of boys and 11.4 % (time 2: 5.9 %) of girls never used the social network site. Correlations between the measures are provided in Table 2.

**Table 1:** Descriptive statistics

	Social network site use				Peer appearance-related feedback				Body dissatisfaction			
	Time 1		Time 2		Time 1		Time 2		Time 1		Time 2	
	Girls	Boys	Girls	Boys	Girls	Boys	Girls	Boys	Girls	Boys	Girls	Boys
Mean	2.79***	2.03***	3.03***	2.19***	0.59**	0.47**	0.70***	0.47***	1.53**	1.38**	1.52**	1.37**
*SD*	1.42	1.56	1.17	1.46	0.60	0.53	0.63	0.54	0.65	0.69	0.67	0.62
Minimum	0	0	0	0	0	0	0	0	0	0	0	0
Maximum	4	4	4	4	3.25	3.25	3.25	3.75	4	4	4	4

Social network site use represents the frequency with which adolescents visit the social network site (0 = *never*, 4 = *always*). Peer appearance-related feedback represents how often peer appearance-related feedback is received from friends (0 = *never,* 4 = *very often*). Body dissatisfaction represents how satisfied respondents are with different appearance attributes (0 = *very satisfied,* 4 = *very dissatisfied*)

* *p* < .05 ** *p* < .01 *** *p* < .001 (two-tailed significance levels for differences in means between boys and girls)

**Table 2:** Zero-Order correlations

	Social network site (SNS) use		Peer appearance-Related feedback		Body dissatisfaction	
	Time 1	Time 2	Time 1	Time 2	Time 1	
SNS use						
Time 1	–					
Time 2	.58*** (.55/.53)	–				
Peer appearance-related feedback						
Time 1	.15*** (.20/.06)	.10* (.09/.06)	–			
Time 2	.18*** (.15/.12)	.14** (.09/.10)	.36*** (.31/.38)	–		
Body dissatisfaction						
Time 1	.05 (.01/.03)	.07 (.01/.05)	.07 (.07/.04)	.04 (.07/−.05)	–	
Time 2	.11* (.13/.05)	.08* (.00/.10)	.02 (.02/.00)	.08 (.09/.02)	.57*** (.52/.60)	

Correlations (Kendall’s tau-a converted to an approximation of Pearson’s *r* using Greiner’s relation) for the total sample and for boys and girls (respectively) in brackets. Social network site (SNS) use represents the frequency with which adolescents visit the social network site (0 = *never*, 4 = *always*). Peer appearance-related feedback represents how often peer appearance-related feedback is received from friends (0 = *never,* 4 = *very often*). Body dissatisfaction represents how satisfied respondents are with different appearance attributes (0 = *very satisfied,* 4 = *very dissatisfied*)

* *p* < .05 ** *p* < .01 *** *p* < .001 (two-tailed, for total sample only)

### Effect of Social Network Site Use on Body Dissatisfaction

The first hypothesis predicted that as adolescents use social network sites more frequently, their body dissatisfaction would increase. To test this hypothesis rigorously, we modeled the hypothesized influence of hypothesis 1, namely the effect of social network site use at time 1 on body dissatisfaction at time 2 in a structural equation model, and controlled for body dissatisfaction at time 1, as outlined in the “Methods” section. To test the alternative direction of effects, we also modeled the influence of body dissatisfaction at time 1 on social network site use at time 2. The resulting model (see Fig. 1) achieved an excellent fit, χ^2^(*df* = 17, *N* = 604) = 15.76, *p* = .54, CFI = 1.00, RMSEA = .00 (90 % CI .00/.03). Frequency of social network site use positively and significantly predicted body dissatisfaction, β = .10, *B* = .04, *SE* = .01*, p* = .008. The bootstrap bias–corrected and accelerated 95 % confidence interval (Bt bca 95 % CI) ranged from .01 to .06. This confidence interval does not include zero, which indicates statistical significance. Body dissatisfaction at time 1 did not predict social network site use at time 2 in this model, β = .04, *B* = .09, *SE* = .08*, p* = .271 (Bt bca 95 % CI −.07/.27). Regression analyses with heteroscedasticity-consistent standard errors (hc3) also showed that social network site use at time 1 positively predicted body dissatisfaction at time 2, *B* = .04, *SE*_*HC3*_ = .02*, p* = .008 (95 % CI .01/.07). The findings thus supported H1, showing that social network site use predicts body dissatisfaction, but body dissatisfaction does not predict social network site use.^1^

### Effect of Social Network Site Use on Peer Appearance-Related Feedback

Hypothesis H2b predicted that as adolescents use social network sites more frequently, their received peer appearance-related feedback would increase. To test this hypothesis we modeled the figure shown in Fig. 2. This model achieved good fit, χ^2^(*df* = 33, *N* = 604) = 72.01, *p* = .000, CFI = .98, RMSEA = .04 (90 % CI .03/.06). Frequency of social network site use at time 1 positively and significantly predicted peer appearance-related feedback at time 2, β = .10, *B* = .04, *SE* = .02*, p* = .016 (Bt bca 95 % CI .00/.07). Peer appearance-feedback at time 1 did not predict social network site use at time 2 in this model, β = .03, *B* = .07, *SE* = .09*, p* = .475 (Bt bca 95 % CI −.11/.26). Regression analyses with heteroscedasticity-consistent standard errors (hc3) also revealed that social network site use at time 1 positively predicted appearance-related feedback from peers at time 2, *B* = .04, *SE*_*HC3*_ = .02*, p* = .010 (95 % CI .01/.07). The findings thus supported H2a, showing that social network site use predicts peer appearance-related feedback, but appearance-related feedback does not predict social network site use.^2^

### Effect of Peer Appearance-Related Feedback on Body Dissatisfaction

Hypothesis H2b predicted that as adolescents receive peer appearance-related feedback more frequently, their body dissatisfaction would increase. To test this hypothesis we modeled the figure shown in Fig. 3. This model achieved good fit, χ^2^(*df* = 74, *N* = 604) = 144.95, *p* = .000, CFI = .98, RMSEA = .04 (90 % CI .03/.05). Frequency of peer appearance-related feedback at time 1 did not predict body dissatisfaction at time 2, β = .02, *B* = .02, *SE* = .04*, p* = .706 (Bt bca 95 % CI −.07/.12). Body dissatisfaction at time 1 also did not predict peer appearance-related feedback at time 2 in this model, β = .01, *B* = .01, *SE* = .04*, p* = .768 (Bt bca 95 % CI −.08/.14). In the regression analyses with heteroscedasticity-consistent standard errors (hc3), peer appearance-related feedback at time 1 also did not predict body dissatisfaction at time 2, *B* = .00, *SE*_*HC3*_ = .04*, p* = .963 (95 % CI −.08/.08). H2b was thus rejected.^3^

### Mediation Model

The second set of hypotheses together stated that social network site use would predict increased peer appearance-related feedback (H2a), which in turn would predict increased body dissatisfaction (H2b), and therefore frequency of social network site use would indirectly lead to body dissatisfaction through peer appearance-related feedback (H2c). The second set of hypotheses was also tested in the AMOS model displayed in Fig. 4, in which the effects of social network site use (time 1) on peer appearance-related feedback (time 1 and time 2), and of peer appearance-related feedback (time 1) on body dissatisfaction (time 2) were modeled, in addition to the autoregressive paths. This model yielded a good fit, χ^2^(*df* = 98, *N* = 604) = 180.88, *p* = .000, CFI = .98, RMSEA = .04 (90 % CI .03/.05).

In line with H2a and the cross-lagged analysis (Fig. 2), the effect of social network site use at time 1 on peer appearance-related feedback at time 1 was positive and significant, β = .11, *B* = .04, *SE* = .02*, p* = .023 (Bt bca 95 % CI .00/.07) as was the effect of social network site use at time 1 on peer appearance-related feedback at time 2, β = .10, *B* = .04, *SE* = .02*, p* = .022 (Bt bca 95 % CI .00/.07). Contrasting H2b and in line with the cross-lagged analysis (Fig. 3), the effect of peer appearance-related feedback (time 1) on body dissatisfaction (time 2) was not significant, β = .02, *B* = .02, *SE* = .04*, p* = .619 (Bt bca 95 % CI −.06/.12). The indirect effect of social network site use (time 1) on body dissatisfaction (time 2) through peer appearance-related feedback (time 1) (H2c) was also not significant, β = .00, *B* = .00, *SE* = .00*, p* = .483 (Bt bca 95 % CI .00/.01). The indirect effect hypothesized under H2c was thus not supported.^4^

### Moderation by Gender

The third set of hypotheses, predicting that the effects specified under H1 and H2a–b would be stronger among girls, was tested using multiple group analyses with gender as the grouping variable. To test H3a, predicting that the positive effect of social network site use on body dissatisfaction would be stronger among girls than boys, we first compared the model testing H1 (Fig. 1) with a model in which we constrained the path from social network site use (time 1) to body dissatisfaction (time 2). When the fits of the constrained and the unconstrained model differ significantly, the focal influence of the constrained path differs significantly between groups. This constrained model did not yield a significantly different fit than the unconstrained model, χ^2^(*1, N* = 604) = 1.85, *p* = .174, TLI_change_ = .00. This suggests that the effect of social network site use on body dissatisfaction was not moderated by gender, contrasting H3a.

We then compared the model testing H2a (Fig. 2) with a partly constrained model in order to test H3b, which noted that the positive effect of social network site use on peer appearance-related feedback would be stronger among girls than among boys. We, therefore, constrained the path from social network site use (time 1) to peer appearance-related feedback (time 2). This constrained model did not yield a significantly different fit than the unconstrained model, χ^2^(*1, N* = 604) = .90, *p* = .343, TLI_change_ = .00. This suggests that the effect of social network site use on peer appearance-related feedback was not moderated by gender. H3b was thus not supported.

To test H3c, which predicted that the positive effect of peer appearance-related feedback on body dissatisfaction would be stronger among girls, we constrained the path from peer appearance-related feedback (time 1) to body dissatisfaction (time 2) in Fig. 3. This constrained model did not have a significantly different fit than the unconstrained model, χ^2^(*1, N* = 604) = 2.57, *p* = .109, TLI_change_ = .00. Contrary to the predictions of H3c, the effect of peer appearance-related feedback on body dissatisfaction was thus not stronger among girls than among boys.

## Discussion

The current study focuses on the impact of social network sites on adolescent boys’ and girls’ body image. The popularity of social network sites among adolescents (Lenhart and Madden 2007; Lenhart et al. 2010; SPOT 2012) and the centrality of physical appearance on these websites (Ringrose 2011; Siibak 2009) have led to concerns regarding their potential negative impact on adolescent body image (Tiggemann and Miller 2010; Tiggemann and Slater 2013). Previous research has established correlations between social network site use and body dissatisfaction among adolescent girls aged 13–18 (Tiggemann and Miller 2010; Tiggemann and Slater 2013). However, questions remained regarding the causal direction of this relationship, the mechanism underlying this relationship, and whether this relationship pertains to boys to the same extent as to girls.

The present study addressed these questions. Regarding the question of temporal direction, this longitudinal study shows that more frequent social network site use predicted increased body dissatisfaction among adolescents 18 months later but body dissatisfaction did not predict social network site use. With respect to the question about mechanisms underlying this relationship, we investigated peer appearance-related feedback as a potential mediator. We found that social network site use predicted more frequent reception of peer appearance-related feedback. However, peer appearance-related feedback did not predict body dissatisfaction. Appearance-related feedback, thus, did not mediate the effect of frequency of social network site use on body dissatisfaction. Regarding the question about the role of gender, boys were affected by social network site use in the same manner and to the same extent as girls. These findings have several theoretical and practical implications and offer useful insights for future research.

In terms of theoretical implications, the results of the current study shed new light on the nature of sociocultural influences on adolescent body image. The tripartite influence model (Thompson et al. 1999b) has considered parents, peers, and mass media as influences on body image. Our finding that social network site use augmented body dissatisfaction suggests that social network site use may be an additional sociocultural channel that influences adolescent body image. This influence may partly overlap with, or resemble, the influences from peers and mass media outlined in the tripartite model (Thompson et al. 1999b), but may also differ. Future research should further investigate the ways in which social network sites impact adolescent body image and focus on the extent to which this impact resembles, or differs from, the influences of mass media, parents, and peers.

The current study has explored a way in which social network sites may exert sociocultural influence on body image that is outlined in the tripartite model, namely through peer influence. As expected, more frequent use of social network sites predicted increased reception of appearance-related feedback from peers. However, we found that appearance-related feedback from peers did not predict body dissatisfaction over time. This finding is not in line with the tripartite model’s notion that peer influence to conform to appearance ideals leads to body dissatisfaction (Thompson et al. 1999b). One explanation for our findings may be that the impact of received peer appearance-related feedback on body dissatisfaction depends on the type and the valence of this feedback, at least among females aged 18 to 25 (Herbozo and Thompson, 2006). The current measure of peer appearance-related feedback did not distinguish different types of feedback. Receiving a mean comment about body weight from your best friend may have different effects on body image than a classmate providing you with tips to make your lips look fuller. Moreover, adolescents in the current sample on average experienced the type of peer influence assessed in the current study never to sometimes. As a result, the current study cannot assert definite conclusions about whether or not social network sites exert their pressures on body image through peer influence and future research is this area needed.

The lack of support for peer appearance-related feedback as an underlying mechanism as well as the low frequency of peer appearance-related feedback (Table 1) may also suggest that there are other, potentially more common, forms of sociocultural influence that mediate the effects of social network site use on body dissatisfaction. Studies should investigate whether and how the opportunities offered by new media to present the own body, to gain public feedback on one’s appearance, and to scrutinize the bodies of others (Meier and Gray 2013; Ringrose 2011; Siibak 2009) affect the body image of its users in ways similar and different to face-to-face peer interactions and mass media exposure. As a result, the tripartite model (Thompson et al. 1999b) may be extended or adapted to incorporate ways in which body image is influenced by creating, sharing and responding to appearance-related content online.

An alternative explanation for the lack of support for the effect of peer appearance-related feedback on body dissatisfaction is that this effect may be stronger among, or confined to, a specific group of adolescents. Previous research has shown differential susceptibility regarding mass media influences on body image (e.g., Groesz et al. 2002; Stice et al. 2001). In the same way, peer appearance-related feedback as well as social network site use may also particularly influence the body image of certain vulnerable adolescents. In this respect, age and developmental characteristics could be relevant individual difference factors to consider (Bartlett et al. 2008; Groesz et al. 2002). The age range of the current study (11–18) was broad and sociocultural influences on body image may differ for adolescents at different ages and developmental stages (Bartlett et al. 2008; Groesz et al. 2002). Future research should thus identify potentially vulnerable groups in order to fully understand the impact of sociocultural influences, including peer appearance-related feedback and social network site use, on body image.

In addition to individual and developmental differences, cultural differences should also be taken into account when interpreting the current findings. For example, the finding that the relationships between social network site use, peer appearance-related feedback, and body dissatisfaction applied to the girls and boys to the same extent and in the same way may not generalize from our Dutch sample to adolescents in other countries. The Netherlands is considered to be a so-called feminine society, in which gender differences are less pronounced than in more masculine societies such as the US (Hofstede 1998). Therefore, a similar study in other countries may lead to different conclusions regarding the role of gender in body image.

Cultural context and other characteristics of our sample are also important to take into account with respect to other aspects of our findings. The adolescents in the current sample were more satisfied than dissatisfied with their bodies on average, with the mean level of body dissatisfaction being halfway between “not satisfied-not unsatisfied” and “quite satisfied.” Moreover, the vast majority of adolescents were in the low or normal BMI range. In the current sample, 91 % of adolescents reported a BMI under 25 and 52 % reported a BMI under 20. Furthermore, on average the current sample never or sometimes received appearance-related feedback from friends. While this points to relatively healthy patterns in youth’s relationships with their bodies among the adolescents in the current Dutch sample, the degree of body dissatisfaction and reception of peer appearance-related feedback may be more troublesome among adolescents in other countries or among certain potentially underrepresented Dutch subgroups. For example, it is documented that, among adult women, body dissatisfaction is lower in Western Europe than in North and South America (Swami et al. 2010). These cross-cultural differences may also apply to adolescents and present an important contextualization of our results. A replication of the current study’s findings in other samples is thus needed to see if these findings generalize to other populations.

The current study has a number of shortcomings that future research can improve on. Future research in this area would benefit from experimental approaches in order to rigorously establish causality. The current study, with its two-wave panel design, can shed first light on the causal direction of relationships established in previous cross-sectional research. However, the current design does not have the same internal validity as an experimental design. Another shortcoming of our study refers to the investigation of hypothesized mediation. Although the current design offers a more thorough approach at establishing the temporal order of mediated effects than cross-sectional designs, a three-wave survey would have been preferable. A final limitation of the current study is the quality of the measurement of social network site use. This measure consisted of only one item and measured the general use of only one social network site, which was very popular in the Netherlands when the study was conducted, but has declined in popularity since (Newcom Research and Consultancy 2012). As adolescents keep switching from one online platform to another, a recommendation for future research is to investigate the impact of activities that are not specific to one platform or to platforms at one moment in time.

In terms of practical implications, our study suggests that adolescents who report high levels of body dissatisfaction or who are at a greater risk for developing body image problems may benefit from interventions or guidelines to decrease the negative impact of social network site use on body image. Such interventions could be beneficial in the same way as some interventions seem effective at decreasing the negative effects of exposure to beauty ideals in the mass media on body image among adolescents aged 13–15 and young college-age women (Wilksch and Wade 2010; Yamamiya et al. 2005). Our finding that social network sites impact boys’ body image to the same extent as girls’ body image suggests that both boys and girls may benefit from such interventions. However, it is important to note that, in the current sample, girls did experience more peer appearance-related feedback and were more dissatisfied with their bodies than boys, although the differences were not extreme (see Table 1). In order to develop interventions and implement these effectively, we need to increase our understanding of how social network site use impacts body image, which specific social network site activities affect body image, among which adolescents this effect occurs most strongly, and under which conditions the effects come about.

## Conclusion

This study offers a number of insights into the role that social network sites have come to play in adolescents’ development and wellbeing. Previous research has shown that these increasingly popular websites (Lenhart et al. 2010; Lenhart and Madden 2007; SPOT 2012) impact adolescent development in several areas, such as adolescents’ relationships and self-esteem (Gentile et al. 2012; Valkenburg et al. 2006). The current study has investigated whether and how social network sites also impact adolescent girls’ and boys’ body image—a crucial aspect of adolescent development and wellbeing (Markey 2010).

The current study contributes to the scarce knowledge about the relationships between social network site use and body image. Previous research has offered an initial indication that the use of social network sites is related to adolescent body image (Tiggemann and Miller 2010; Tiggemann and Slater 2013). In a number of ways, the current longitudinal study builds on these correlational studies among girls aged 13–18 (Tiggemann and Miller 2010; Tiggemann and Slater 2013). First, this study offers initial information about the causal direction of this association by showing that more frequent social network site use predicts increased body dissatisfaction over time. Second, the present study shows that social network site use predicts more frequent reception of peer appearance-related feedback. However, peer appearance-related feedback did not explain the effect of social network site use on body image because peer appearance-related feedback did not predict body dissatisfaction. Other mechanisms may be at play and/or only certain appearance-related feedback may lead to body dissatisfaction, only in certain situations or only among certain individuals. Third, this study shows that social network site use impacts the body image and reception of peer appearance-related feedback among boys to the same extent as among girls.

In this study, we offer a number of insights and suggestions that future research on adolescents’ body image can build on. Specifically, more research is necessary to replicate the findings in other groups and countries, to assess which social network site activities impact body image and through which mechanisms, and to determine in which situations and among which adolescents the effects of social network site use on body image may be stronger or weaker. Nevertheless, the current study offers important evidence that social network site use poses a risk to adolescent boys’ and girls’ body image. Researchers, parents, and practitioners should aim to understand and try to counter these negative effects.

## Footnotes

1 When we reran the model testing hypothesis 1 (Fig. 1) with control variables, the model again achieved good fit, χ²(*df* = 37, *N* = 524) = 50.92, *p* = .063, CFI = .99, RMSEA = .03 (90 % CI .00/.04). Frequency of social network site (SNS) use at time 1 again positively and significantly predicted body dissatisfaction at time 2, β = .11, *B* = .04, *SE* *=* .02*, p* = .008 (Bt bca 95 % CI .01/.08), again supporting hypothesis 1. Body dissatisfaction at time 1 again did not predict SNS use at time 2, β = .03, *B* = .06, *SE* *=* .09*, p* = .491 (Bt bca 95 % CI −.16/.25). Regarding the control variables, body dissatisfaction at time 2 was significantly predicted by higher BMI at time 1, β = .09, *B* = .02, *SE* *=* .01*, p* = .035 (Bt bca 95 % CI −.00/.03), but not by the other control variables. SNS use at time 2 was predicted significantly only by lower age, β = −.10, *B* = −.08, *SE* *=* .03*, p* = .018 (Bt bca 95 % CI −.14/−.01).

2 When we reran the model testing hypothesis 2a (Figure 2) with control variables, the model again achieved good fit, χ²(*df* = 70, *N* = 524) = 133.14, *p* = .000, CFI = .97, RMSEA = .04 (90 % CI .03/.05). Frequency of social network site (SNS) use at time 1 again positively and significantly predicted peer appearance-related feedback at time 2, β = .11, *B* = .04, *SE* *=* .02*, p* = .015 (Bt bca 95 % CI .01/.08), again supporting hypothesis H2a. Peer appearance-feedback at time 1 again did not predict SNS use at time 2 in this model, β = .02, *B* = .04, *SE* *=* .10*, p* = .666 (Bt bca 95 % CI −.16/.22).

3 When we reran the model testing hypothesis 2b (Figure 3) with control variables, the model again achieved good fit, χ²(*df* = 124, *N* = 524) = 213.23, *p* = .000, CFI = .98, RMSEA = .04 (90 % CI:.03/.05). Peer appearance-related feedback at time 1 again did not predict body dissatisfaction at time 2, β = .04, *B* = .04, *SE* *=* .05*, p* = .342 (Bt bca 95 % CI −.04/.16), again not supporting H2b. Body dissatisfaction at time 1 also again did not predict peer appearance-related feedback at time 2 in in this model, β = .02, *B* = .02, *SE* *=* .05*, p* = .636 (Bt bca 95 % CI −.09/.15). Body dissatisfaction at time 2 was again predicted by BMI, β = .09, *B* = .02, *SE* *=* .01*, p* = .028 (Bt bca 95 % CI .00/.03).

4 The model in Figure 4 with added covariates also yielded a good fit, χ²(*df* = 148, *N* = 524) = 248.23, *p* = .000, CFI = .98, RMSEA = .04 (90 % CI .03/.04). The indirect effect of social network site use on body dissatisfaction (H2c) was again not significant, β = .01, *B* = .00, *SE* *=* .00*, p* = .186 (Bt bca 95 % CI .00/.01).
